# Targeting epidermal lipids for treatment of Mendelian disorders of cornification

**DOI:** 10.1186/1750-1172-9-33

**Published:** 2014-03-07

**Authors:** Dimitra Kiritsi, Franziska Schauer, Ute Wölfle, Manthoula Valari, Leena Bruckner-Tuderman, Cristina Has, Rudolf Happle

**Affiliations:** 1 Department of Dermatology, Medical Center-University of Freiburg, 79104 Freiburg, Germany; 2 Department of Dermatology, Agia Sofia Children’s Hospital, Athens, Greece

**Keywords:** Cholesterol, Mosaicism, Ichthyosis, Simvastatin, CHILD nevus, *NSDHL* mutations

## Abstract

**Background:**

Inherited ichthyoses or Mendelian disorders of cornification (MeDOC) are clinically heterogeneous disorders with high unmet therapeutic needs, which are characterized by skin hyperkeratosis and scaling. Some MeDOC types are associated with defects of the epidermal lipid metabolism, resulting in perturbed barrier permeability and subsequent epidermal hyperplasia, hyperkeratosis and inflammation. An example is the CHILD (congenital hemidysplasia with ichthyosiform nevus and limb defects) syndrome, an X-linked dominant multisystem MeDOC caused by mutations in the *NSDHL* (NAD(P)H steroid dehydrogenase-like protein) gene, which is involved in the distal cholesterol biosynthetic pathway. The skin manifestations of the CHILD syndrome have been attributed to two major mechanisms: deficiency of cholesterol, probably influencing the proper corneocyte membrane formation, and toxic accumulation of aberrant steroid precursors.

**Methods:**

Here we addressed the efficacy of an ointment containing cholesterol and simvastatin, an agent inhibiting endogenous cholesterol synthesis in a compassionate-use treatment of three patients with CHILD syndrome. To test the specificity of this therapeutic approach, we applied the same topical treatment to two patients with other types of MeDOC with disturbed skin lipid metabolism.

**Results:**

The therapy with simvastatin and cholesterol was highly effective and well-tolerated by the CHILD syndrome patients; only lesions in the body folds represented a therapeutic challenge. No improvement was noted in the patients with other types of MeDOC.

**Conclusions:**

This therapy is inexpensive and accessible to every patient with CHILD syndrome, because both simvastatin and cholesterol are available worldwide. Our data provide initial evidence of the specificity of the therapeutic effect of the simvastatin-cholesterol ointment in CHILD syndrome in comparison to other types of MeDOC.

## Background

Inherited ichthyoses or Mendelian disorders of cornification (MeDOC) are a large group of clinically heterogenous disorders characterized by more or less diffuse hyperkeratosis and scaling of the skin, and high unmet therapeutic needs [1]. The genetic basis is diverse with autosomal dominant, recessive and X-linked types [2,3]. So far, only topical modes of treatment with ointments containing keratolytics and emollients, as well as oral retinoids are available [4]. These approaches are only symptomatic and do not address the specific pathogenesis of each MeDOC type. The development of more specific therapies depends on the elucidation of the underlying gene defect and understanding of the disease pathogenesis [5].

Epidermal differentiation is a tightly regulated process where multiple players are involved. The cohesion of the outermost layer of the epidermis, the stratum corneum, is assured through the embedment of anucleate keratinocytes, called corneocytes in a lipid-enriched extracellular matrix. This organization resembles a wall, with the bricks being represented by the corneocytes and the mortar by the epidermal lipids, cholesterol, ceramides and free fatty acids [6]. Mutations in genes connected to the lipid metabolism of the skin result in perturbed barrier permeability and subsequently in epidermal hyperplasia, hyperkeratosis, inflammation and barrier dysfunction. The CHILD (congenital hemidysplasia with ichthyosiform nevus and limb defects) syndrome is an X-linked dominant multisystem MeDOC caused by mutations in the *NSDHL* (NAD(P)H steroid dehydrogenase-like protein) gene, which is involved in the distal cholesterol biosynthetic pathway [7]. The skin phenotype comprises erythrodermic ichthyosis restricted to the one side of the body, with characteristic wax-like scales. Further, this devastating disease is associated with ipsilateral hypoplasia of the body, affecting the development of brain, skeletal structures and viscera [8]. Two major mechanisms have been suggested to be responsible for the cutaneous manifestations: the cholesterol deficiency itself, which probably influences the proper formation of the corneocyte membrane, and the toxic accumulation of the steroid precursors [5].

In this study we addressed the efficacy of the topical application of an ointment containing cholesterol and simvastatin, an agent inhibiting the endogenous cholesterol synthesis, in a compassionate-use treatment of three patients with CHILD syndrome. Initially, a similar formulation consisting of a lotion containing the cholesterol lowering agent lovastatin and 2% cholesterol was successfully applied in two patients with CHILD syndrome [9]. In addition, Seeger et al. recently mentioned to have initiated this pathogenesis-based combination topical therapy in other CHILD syndrome patients with positive responses [10]. Here we tested the effects of simvastatin and the specificity of this pathogenesis-based therapy and applied the same topical treatment to one patient with autosomal recessive congenital ichthyosis and one with X-linked recessive ichthyosis.

## Methods

The ointment was produced in analogy to a lotion containing the cholesterol lowering agent lovastatin and 2% cholesterol as described previously [6,9,11,12]. As an improvement of the formulation we employed 2% cholesterol combined with simvastatin instead of lovastatin, since it has a higher absorption capacity [13] and is less expensive. To optimize the skin tolerance and compatibility, we tested several excipients and selected Unguentum Cordes®, because simvastatin and cholesterol powder could be directly mixed with it. Unguentum Cordes® is an amphiphilic, hypoallergenic vehicle (INCI: macrogol stearate 400, glycol. monostearate 44-50, sorbitan monostearate and petroleum jelly) which is free of lanolin, fragrance and preserving agents, and is therefore applicable for very sensitive skin. This ointment comprising 2% simvastatin and 2% cholesterol in Unguentum Cordes® will be referred to as S/C ointment.

For morphological analysis of the skin, immunohistochemistry was performed on frozen tissue sections by use of the primary antibodies against filaggrin FLG01 (Abcam, Cambridge, UK), Ki67 (clone MIB-1), CD3 (clone F7.2.38), CD4 (clone 4B12), CD8 (clone C8/144B), CD20 (clone L26) and CD68 (clone KP1), and the AEC (3-amino-9-ethylcarbazole) system (all from DAKO, Hamburg, Germany) and haematoxylin as a counter stain. Information on the patients and the disease-causing mutations is summarized in Table 1. *NSDHL* mutation analysis results have been described before [7].

**Table 1:** Patients treated with the S/C ointment in this study

**Patient no.**	**Type of MeDOC**	**Mutation analysis (gene, mutation on cDNA and protein level)**	**Treatment success**
**Age (years)**			
**Origin**			
1	CHILD syndrome	*NSDHL:* c.613G>A, p.G205S (heterozygous)	Yes
18			
German			
2	CHILD syndrome	*NSDHL:* c.628C>T, p.Q210X (heterozygous)	Yes
38			
German			
3	CHILD syndrome	*NSDHL:* c.262C>T, p.R88X (heterozygous)	Yes
29			
Egyptian			
4	X-linked recessive ichthyosis	Deletion of *STS* (hemizygous)	No
16			
German			
5	Autosomal recessive congenital ichthyosis	*NIPAL4*: c.527C>A; p.A176D (homozygous)	No
13			
Albanian			

## Results and discussion

**Figure 1:**
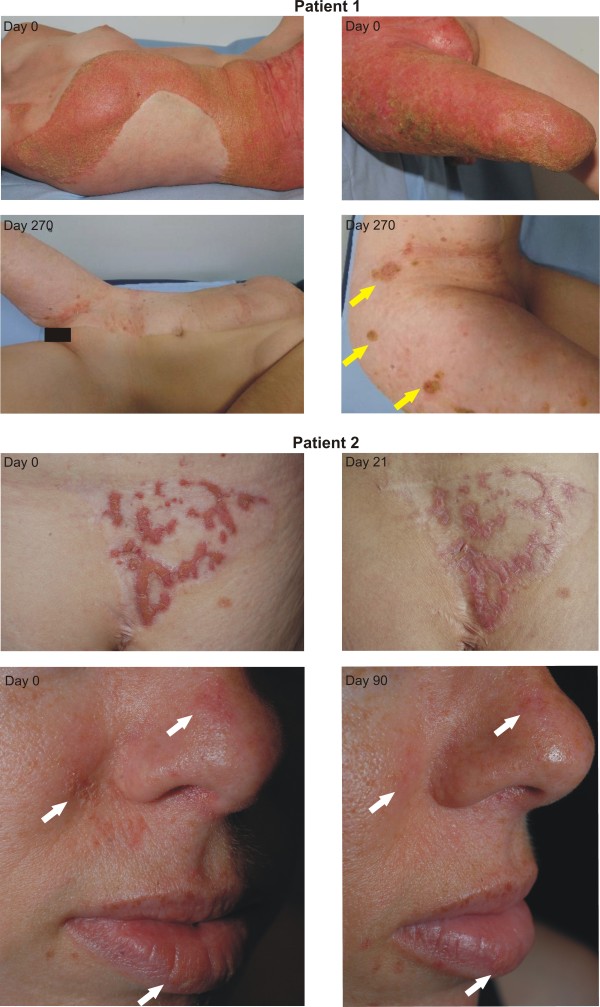
**Clinical features of CHILD nevus before (day 0) and after treatment with the S/C ointment.** In the upper panels patient 1 is shown with a sharply demarcated ichthyosiform nevus on the right side of her body and single verrucous and exudative crusty lesions on the ipsilateral buttock and inguinal fold. After application of the S/C ointment for 9 months the skin lesions have almost completely cleared, except for isolated verrucous lesions that persisted after therapy (yellow arrows). The lower panels show photographs of patient 2 who had erythematous scaly lesions on the right side of her buttock and smaller lesions on the face. The patient applied the S/C ointment twice daily. Note significant improvement of the lesions on the buttocks after 3 weeks and of her facial lesions within 3 months.

Patient 1 was an 18-year-old woman with CHILD syndrome and a sharply demarcated ichthyosiform nevus covering the right side of her body (Figure 1). Single verrucous and exudative crusty lesions were present on the ipsilateral buttock and inguinal fold covering about 20% of the total body surface. Apart from missing right arm and right lower leg she was healthy. Initially, the S/C ointment was applied twice daily only on the abdominal skin. Already after 2 days substantial improvement was noted and after 4 weeks the therapeutic regime was extended to all affected skin regions, resulting in almost complete remission of the erythema and scaling (Figure 1, upper panels). The patient is in follow-up since more than 18 months and currently, she applies the S/C ointment once every 3 weeks to maintain the remission (around 50 gr/month). Her life quality improved dramatically with a DLQI (Dermatology Life Quality Index) score of 15 before treatment to a score of 3, nine weeks after. She can now regularly participate in outdoor and sport activities, e.g. swimming, and is able to wear leg prosthesis. The right groin and perivulvar region showed a slower improvement because of pre-existing oozing lesions. Swabs revealed colonization with Gram-positive and -negative bacteria. In these regions clinical improvement was only achieved after application of oral antibiotics and topical antiseptic powders to reduce the discharge. Isolated verrucous lesions (Figure 1, yellow arrows) persisted after therapy and had to be excised. Histologic examination revealed features of verruciform xanthoma, which represents a characteristic sign of CHILD nevus mainly in body folds [14]. Throughout the follow-up period laboratory examinations including blood count, liver and kidney values, electrolytes and blood lipids were regularly performed and displayed no abnormalities.

Patient 2 with CHILD syndrome was a 38-year-old woman with erythematous scaly lesions on the right side of her back and smaller lesions involving her face and ipsilateral skeletal defects (for details see case 2 in [15]. In the past, her CHILD nevus had been dermabraded and covered with split skin obtained from a contralateral unaffected donor region [16]. This approach resulted in a grafted area free of ichthyotic skin manifestations, besides a triangular region of about 6 × 6 cm on the buttock (about 2% of the body surface), where the lesions reappeared after three grafting attempts. The patient applied the S/C ointment twice daily and noted significant reduction of scaling and itching already after one week, and substantially improved appearance after three weeks (Figure 1, lower panels). She used the S/C ointment to treat her facial lesions with similar satisfactory results (Figure 1). She also reported a considerable improvement of her life quality (the DLQI score dropped from 8 to 1 within 9 weeks of treatment), especially since her skin lesions were not itchy anymore and her daily activities less exhausting.

The third patient with CHILD syndrome was a 29-year-old man with wax-like scales following the Blaschko lines on the right side of his chest and right arm, and erythematous, scaly and verrucous lesions on his right lower leg (for details see [17]). After one week of application of the S/C ointment he noted improvement that became more pronounced after four months. Similar to case 1, however, some thick verrucous lesions persisted on the leg. Unfortunately, the patient was lost for follow-up.

**Figure 2:**
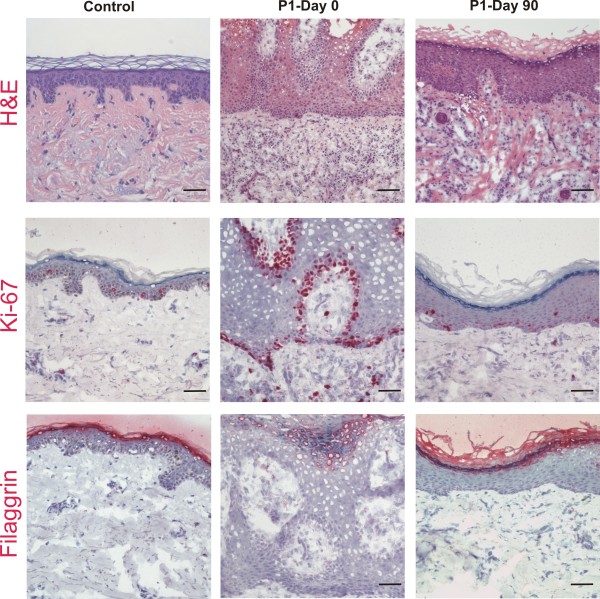
**Morphological analysis of lesional skin of patient 1 before (day 0), and 3 months after initiation of treatment (day 90).** The haematoxylin & eosin (H&E) staining of a skin sample of patient 1 before treatment showed zones of parakeratosis intermingled with areas of orthohyperkeratosis and marked acanthosis. Lymphocytic infiltrates were found in the dermis. Three months after treatment, the skin morphology as evaluated by H&E was comparable to control skin. Similarly, Ki-67 was strongly enhanced and filaggrin showed a disturbed staining pattern before treatment, features that were reverted after application of the S/C ointment. Scale bars: 100 μm.

**Figure 3:**
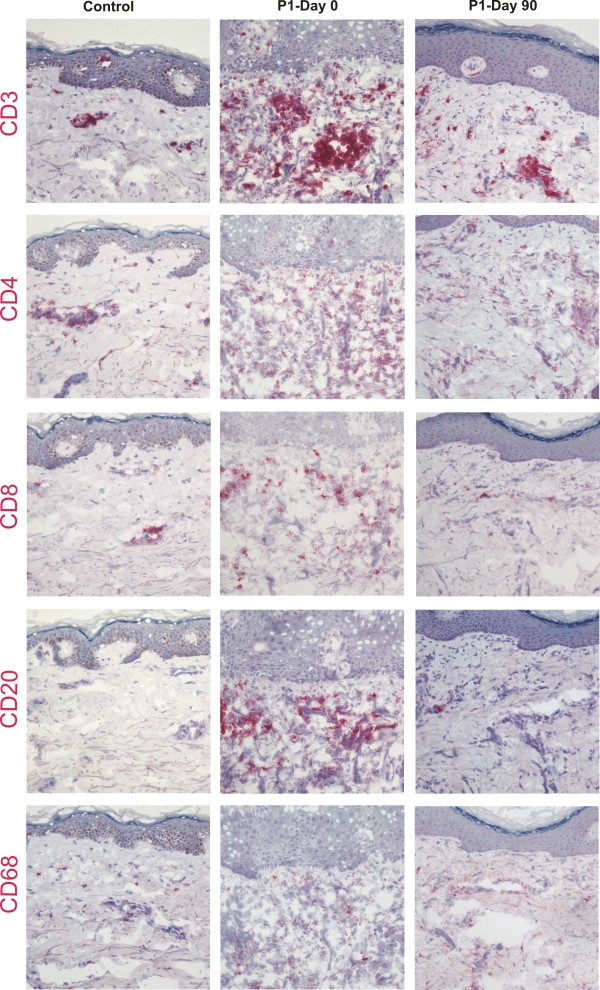
**Evaluation of the inflammatory infiltrate in lesional skin of patient 1 before (day 0) and 3 months after initiation of treatment (day 90).** In the skin biopsy of patient 1 taken at day 0, infiltrates of CD3 positive inflammatory cells were present, as well as CD4, CD8, CD20 or CD68 positive infiltrates. These were significantly reduced after 3 months of treatment.

To assess the effect of the S/C ointment on skin morphology, biopsies were taken from patients 1 and 2 before initiation of the therapy (day 0) and three months after daily application of the S/C ointment (day 90). Before treatment, the affected epidermis of patient 1 was clearly hyperproliferative as visualized with Ki-67 staining, and differentiation was perturbed as shown by the broad staining of filaggrin in the upper epidermal layers (Figure 2). After treatment, the epidermal thickness normalized and the staining patterns of the upper markers were comparable to control skin. In the biopsy taken at day 0 infiltrates of CD3 (mature T-cells) positive inflammatory cells were present, but we also noted CD4 (mainly T-helper cells), CD8 (mainly cytotoxic T-cells), CD20 (B-cells) or CD68 (monocytes/macrophages) positive infiltrates [14], which regressed after treatment (Figure 3). Similar results were obtained for the skin samples of patient 2 (not shown). These data suggest that depletion of abnormal lipid metabolites and replacement with exogenous cholesterol lead to reversion of the hyperproliferative and inflammatory processes in the CHILD nevus.

In order to test the specificity of this therapeutic approach, we applied the S/C ointment in two further patients with other types of MeDOC caused by perturbed lipid metabolism pathways. A 16-year-old boy with X-linked recessive ichthyosis due to a hemizygous deletion of the steroid sulfatase gene (*STS*) presented with greyish scales, especially evident on his neck, upper arms and lower legs (Table 1). He applied the S/C ointment twice daily on the right side of his neck and the right arm for two months, without significant improvement of his skin condition. Finally, a 13-year-old boy with autosomal recessive congenital ichthyosis due to the homozygous *NIPAL4* mutation c.527C > A; p.A176D was treated. Ichthyin, the polypeptide encoded by the *NIPAL4* gene, has been shown to play a physiologic role within the hepoxilin pathway and is therefore essential for the lipid processing in the upper epidermis [18]. Application of the S/C ointment yielded no satisfactory therapeutic outcome. In both patients treatment was discontinued after two months because of insufficient therapeutic response. Nevertheless, patients with other types of MeDOC with abnormal cholesterol/lipid biosynthesis would probably profit from similar topical formulations; how systemic manifestations could be amended remains to be studied in animal models. Patients with Conradi-Hünermann-Happle (CHH) syndrome would almost certainly benefit from the S/C ointment, since the disease pathogenetic mechanism shares the same pathway as CHILD syndrome. However, in CHH the ichthyotic lesions are transient [19,20]. In a mouse model for ichthyosis follicularis with atrichia and photophobia (IFAP) syndrome [21] topical simvastatin improved the skin phenotype [22]. Finally in a patient with Sjögren-Larsson syndrome the application of a lovastatin 2% and cholesterol 2% water solution ameliorated the skin condition [12].

## Conclusions

We employed S/C ointment to treat three patients with CHILD nevus, the cutaneous hallmark of CHILD syndrome. This therapy was highly effective and well-tolerated; a long-term remission of the skin manifestations was obtained. The ointment is inexpensive and accessible to most patients with CHILD syndrome, because both simvastatin and cholesterol are largely available. Lesions involving the body folds represented a therapeutic challenge because of colonization with various microbial strains. Moreover, some recalcitrant lesions showing histological features of verruciform xanthoma had to be excised. Since no improvement was observed in two patients with other types of MeDOC, our data suggest that the therapeutic effect is specific for the CHILD syndrome, and probably closely related disorders with similar defects of cholesterol biosynthesis.

## Abbreviations

CHILD: Congenital hemidysplasia with ichthyosiform nevus and limb defects; DLQI: Dermatology Life Quality Index; MeDOC: Mendelian disorders of cornification; NIPAL4: NIPA-like domain containing 4; NAD(P)H: Steroid dehydrogenase-like protein; S/C ointment: Simvastatin-cholesterol ointment; STS: Steroid sulfatase.

## Competing interests

The authors declare that they have no competing interests.

## Authors’ contributions

DK performed the necessary invasive diagnostic procedures and the experiments. DK, FS, RH and CH are responsible for the follow up of the patients. DK, FS, UW, LBT, CH and RH participated in the interpretation of data. DK and RH designed the study and drafted the manuscript. CH participated in the design and coordination of the study and helped to draft the manuscript. All authors have read and approved the final manuscript.
